# An improved microtiter plate assay to monitor the oxidative burst in monocot and dicot plant cell suspension cultures

**DOI:** 10.1186/s13007-016-0110-1

**Published:** 2016-01-26

**Authors:** Rebecca L. J. Melcher, Bruno M. Moerschbacher

**Affiliations:** Institute for Biology and Biotechnology of Plants, University of Münster, Schlossplatz 8, 48143 Münster, Germany

**Keywords:** Oxidative burst, Monocots, Dicots, Microtiter plate, L-012, Plant defense, Ulvan, Chitosan

## Abstract

**Background:**

A screening method for elicitor and priming agents does not only allow detecting new bioactive substances, it can also be used to understand structure–function relationships of known agents by testing different derivatives of them. This can not only provide new lead compounds for the development of novel, more environment-benign, bio-based agro-chemicals, it may eventually also lead to a better understanding of defense mechanisms in plants. Reactive oxygen species (ROS) are sensitive indicators of these mechanisms but current assay formats are not suitable for multiplex screening, in particularly not in the case of monocot systems.

**Results:**

Here we describe continuous monitoring of ROS in 96-well microtiter plates using the chemiluminescent probe L012, a luminol derivative producing chemiluminescence when oxidised by ROS like hydrogen peroxide, superoxide, or hydroxyl radical that can thus be used as an indicator for these ROS. We were able to measure ROS in both monocot (*Oryza sativa*) and dicot (*Medicago truncatula*) cell suspension cultures and record dose dependencies for the carbohydrate elicitors and priming agents ulvan and chitosan at low substrate concentrations (0.3–2.5 µg/ml). The method was optimized in terms of cell density, L012 concentration, and pre-incubation time. In contrast to the single peak observed using a cuvette luminometer, the improved method revealed a double burst in both cell systems during the 90-min measuring period, probably due to the detection of multiple ROS rather than only H_2_O_2_.

**Conclusion:**

We provide a medium throughput screening method for monocot and dicot suspension-cultured cells that enables direct comparison of monocot and dicot plant systems regarding their reaction to different signaling molecules.

**Electronic supplementary material:**

The online version of this article (doi:10.1186/s13007-016-0110-1) contains supplementary material, which is available to authorized users.

## Background

The first line of defense in plants is the rapid and transient production of reactive oxygen species (ROS) such as superoxide (O_2_^−^) and hydrogen peroxide (H_2_O_2_) during the so-called oxidative burst. Triggered by elicitor recognition through specialized plasma membrane receptors [1], ROS cause oxidative damage to the proteins, nucleic acids and lipids of invading pathogens [2]. They may also help to trigger subsequent defense mechanisms including the synthesis of antimicrobial phytoalexins and hydrolytic enzymes, the construction of defensive barriers such as the lignification of cell walls [3], and the hypersensitive response (HR) [4]. Furthermore, ROS production spreads systemically [5] and induces resistance [6, 7] and immunity [8]. An aspect of induced resistance is the so called priming effect, leading to a faster and stronger response to new threads [9] by accumulation of dormant mitogen-activated protein kinases (MAPKs) [10] and histone modification [11]. Natural compounds that have the ability to induce priming in plants may be developed into novel plant protection agents which strengthen the plants’ own defense potential. Being bio-based, such “agro-biologics” are potentially more environment-benign than current, synthetic agro-chemicals. Elicitor and priming-active biologics can be identified by monitoring ROS in plant tissues or cells treated with them. An interesting group of such agents are carbohydrates which occur not only as exogenous but also as endogenous elicitors. The latter are often referred to as damage associated molecular patterns (DAMPs), the former as pathogen/microbe-associated molecular patterns (PAMPs or MAMPs). Both are often carbohydrates derived from fungal or plant cell walls, such as chitin fragments [12, 13], oligomers and polymers of chitosan [14], oligoglucans [15], or oligopectates [16].

Another polysaccharide that has been shown to be an activator of plant defense [17] and the induction of plant resistance [18] is the sulfated heteropolysaccharide ulvan. It is derived from green macro algae of the genus *Ulva* [19, 20], a so far underexploited biomass with promising properties. As ulvan is not a natural protagonist in plant defense, in contrast to the other carbohydrates mentioned above, a promising approach for the development of novel agro-biologics is to use it as a lead compound, subtly altering its structure and screening for the impact on its ability to induce plant defense mechanisms. As an example, one optimization goal would be to decrease the molecular weight of ulvan, and thus its viscosity, without losing activity to improve the handling in agricultural practice. To this end, a screening assay that is fast and has a reasonable throughput is required.

H_2_O_2_ production can be measured over time in plant tissue extracts using the chemiluminescent reagent luminol combined with the catalyst potassium ferricyanide [21]. A highly sensitive assay based on plant cell suspension cultures has been developed to identify elicitors or priming agents, and to study their modes of action [22]. This assay was originally developed using dicot cells but was later adapted for monocot cells which are much more difficult to handle [23–25]. In each case, luminol and potassium ferricyanide are added to aliquots of the assay medium taken at different time points after elicitation, and H_2_O_2_ is quantified using a cuvette luminometer. The assay typically reveals a characteristic peak of H_2_O_2_ production with an onset 5–10 min after elicitation, reaching a maximum after 20–30 min and returning to pre-stimulation levels within ~90 min.

Although the luminol method is reliable and sensitive, the laborious assay procedure limits the number of samples that can be processed simultaneously. This is a major challenge if a range of different substances need to be compared because they must be split among several measurements. Cell suspension cultures tend to be heterogeneous in terms of growth and behavior, so variable amounts of H_2_O_2_ are produced in different experiments. The use of internal standards can only partially alleviate this problem. We therefore adapted the assay to a 96-well microtiter plate format in order to increase the number of samples that can be processed in parallel allowing the generation of statistically useful datasets.

The oxidative burst in mammalian cells has been measured in microtiter plate assays using luminol [26] and dichlorofluorescein [27, 28]. The latter was also used to develop a microtiter plate assay for tobacco cells showing a gradual increase in fluorescence [29]. In our hands, this assay had a low sensitivity and was unable to reveal dose dependencies when tested against a range of cultured plant cell lines.

The original luminol assay had been used successfully to quantify ROS in extracts of dicot plant tissues [21], in the culture medium of dicot plant cells [22], or in exudates of leaf discs [30]. In the latter case, 96-well microtiter plate assays using luminol [31] or the luminol derivative L012 (8-amino-5-chloro-7-phenylpyrido[3,4-d]pyridazine-1,4(2H,3H)dione) have also been described [32, 33]. However, we were unable to adapt these 96-well microtiter plate assays for monocot leaf discs. The leaf discs showed ROS production, but even the water-treated negative control always reacted, and up to tenfold increased ROS levels occurred randomly without correlation to treatment.

To address this challenge, we modified the luminol-based method described above for the quantification of ROS in aliquots of cell culture suspensions, adapting it to work with both monocot and dicot cells treated with an elicitor of the oxidative burst [23]. Luminol was replaced with the more sensitive L012 [34, 35], a luminol derivative producing chemiluminescence when oxidised by ROS like hydrogen peroxide, superoxide, or hydroxyl radical. It allows continuous monitoring of ROS, as previously shown for infiltrated tobacco leaves [36], *Arabidopsis* roots [37], and leaf discs from dicot species [32].

The assay we developed is suitable for the continuous measurement of the oxidative burst in both monocot and dicot cell suspension cultures, revealing the presence of a ‘double burst’ suggesting that ROS are being produced in a biphasic manner in response to stress.

## Results

The original cuvette luminometer method for the detection of ROS was sensitive but the laborious assay method limited the number of samples that could be processed simultaneously. We therefore adapted the method by transferring it to a microtiter plate luminometer. By replicating the luminol/potassium ferricyanide method used with the cuvette luminometer, we were able to detect a peak at 20 min after ROS initiation, but continuous measurement was not possible because the potassium ferricyanide used for the measurements is toxic, and each sampling point therefore required an additional well.

We changed the luminescent agent from luminol to L012, which does not require potassium ferricyanide as a catalyst and should therefore be more suitable for the continuous measurement of individual wells. In an initial experiment, we were able to measure the oxidative burst of *Medicago truncatula* cell suspension cultures in 96 wells in parallel, and to record the dose dependency of the elicitor ulvan (Additional file 1). The dose dependency was notable in the slope immediately after elicitation, indicating a clear relationship between the concentration of elicitor and the generation of ROS, but the amplitudes of different doses were neither well distinguishable, especially for high elicitor concentrations, nor well reproducible. The most striking difference between the new continuous method and the old aliquot-based method was the distinct appearance of a double burst with peaks at 10–20 and 50 min. We noted that the ROS curve did not fully return to the baseline during the measuring period of 90 min. Prolonged measurement showed a third slow peak after several hours followed by a return to the baseline. This slow, late, and transient production of ROS was also observed in the untreated cells, albeit at a lower level than in elicitor-treated cells (data not shown).

**Fig. 1:**
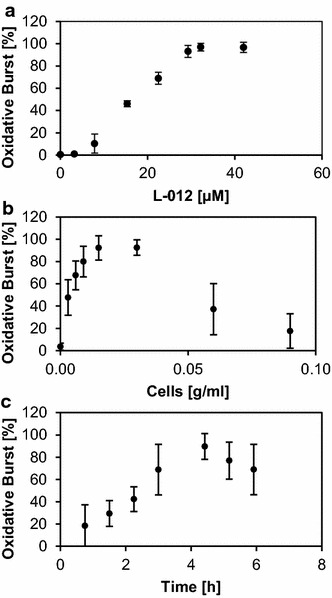
Condition optimization in microtiter plate for dicot suspension cultures of *Medicago truncatula.* Optimization of parameters for the measurement of an oxidative burst in *Medicago truncatula* cell suspension cultures elicited with 0.1 mg/ml ulvan, using a microtiter plate assay format in triplicates: concentration of L012 (**a**), cell density (**b**), and pre-incubation time (**c**). Values are expressed as a percentage of the maximum relative light units (RLUs) measured over a 90-min interval, and represent mean ± SD of three independent experiments

Next, we optimized the L012 concentration, where the dose dependency curve showed sigmoidal behavior with the strongest increase between 6 and 30 µM (Fig. 1a). For further experiments, a L012 concentration of 30 µM was chosen. Addition of L012 induced a small emission of light, possibly due to alkalization of the medium caused by the buffer used to solubilize L012. Therefore, we included the L012 in the assay medium during pre-incubation to exclude interference of elicitor- and L012/pH-induced ROS production. This did not influence the sensitivity of the assay but generated a straight baseline in the untreated negative control cells. We measured independent cell lines pre-treated with L012 in six independent experiments, and the early double peak after elicitation with ulvan was observed in all cases (data not shown). A maximum light output was achieved at a cell density between 0.01 and 0.03 g/ml (Fig. 1b) and with a pre-incubation period in assay medium of 4 h (Fig. 1c). Interestingly, the pre-incubation time of 4 h not only led to the strongest burst, but also to the lowest variability among experiments. The optimal conditions for the assay therefore consisted in 4 h pre-incubation with 9 mg/l L012 and a cell density of 0.015 g/ml. The early double burst was visible in all optimization experiments.

**Fig. 2:**
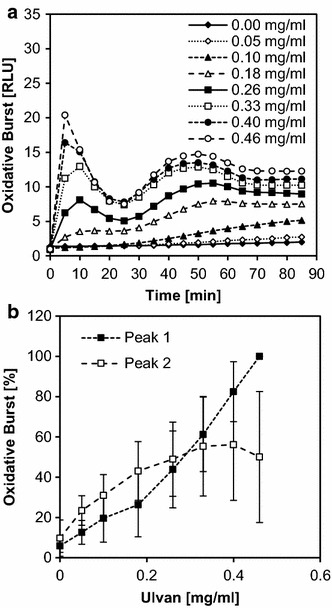
Elicitor dose dependency in the dicot *Medicago truncatula.* Dose dependency of the oxidative burst in *Medicago truncatula* cell suspension cultures elicited with different concentrations of ulvan (0.05–0.46 mg/ml) in triplicates. During the 90-min measuring period, first a peak with high amplitude and short duration was observed followed by a second peak with lower amplitude and longer duration (**a**). Each peak maximum was used to plot dose-dependency curves, in which peak 1 is defined as 0–20 min and peak 2 as 25–60 min (**b**). The data shown in **a** are taken from one experiment which is representative of six independent experiments. The data shown in **b** are mean values ± SD of these six experiments with the maximum relative light units (RLUs) reached in each experiment set to 100 %. P values resulting from a Whitman-rank test were combined and showed that the two curves were significantly different

Using these optimal conditions, we recorded a dose response curve with different concentrations of ulvan as an elicitor in *M. truncatula* cells (Fig. 2a). On purpose, we used ulvan as a weak elicitor, compared to natural elicitors, in order to develop a test system that would allow the identification of candidate molecules that might serve as lead structures for the development of novel elicitor-active substances.

Clear dose dependency was visible at all time points. The first peak occurred between 0 and 30 min after elicitation, reaching a maximum at 10 min, and the second peak followed with a maximum after 50 min. In comparison, the first peak had high amplitude and was of short duration, whereas the second peak showed lower amplitude but lasted twice as long. Over the concentration range measured, the amplitudes of the dose steadily increase for the first peak but described a saturation curve for the second peak, crossing the dose dependency curve of the first peak at ~0.3 mg/ml of ulvan (Fig. 2b). To test for significant difference between the dose response curves of the first and second amplitude, a Mann–Whitney rank test was performed for each pair of data points. Raw data from five experiments performed in triplicates were taken and the combined p value was calculated (0.000036).

The double burst became more obvious as the elicitor concentration increased. The measurement was carried out 20 times in four replicate *M. truncatula* cell lines and the double burst was detected 18 times (90 %) regardless of which cell line was used. The addition of catalase (175 U/ml) reduced the amplitudes of both peaks strongly (Additional file 2) and verified the bursts being ROS dependent.

**Fig. 3:**
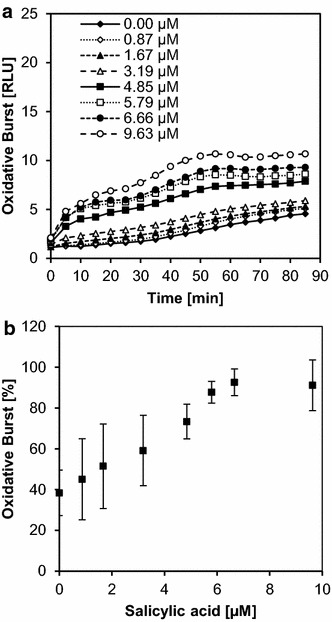
Priming dose dependency in the dicot *Medicago truncatula.* Dose dependency of the oxidative burst in *Medicago truncatula* cell suspension cultures pre-treated with different concentrations of salicylic acid (0.8–9.6 µM) in triplicates, then elicited with ulvan (0.08 mg/ml). During the 90-min measurement period, two peaks can be observed (**a**). The first peak maximum (0–25 min) was used to plot a dose dependency curve (**b**). The curve for the second peak was similar (not shown). The data shown in **a** are taken from one experiment which is representative of three independent experiments. The data shown in **b** are mean values ± SD of these three experiments with the maximum relative light units (RLUs) reached in each experiment set to 100 %

The optimized method allowed us to also set up a priming assay for *M. truncatula* cells, as priming agents are promising lead compounds for plant protection. So far, only few priming agents for *M.truncatula* have been described and we therefore pre-treated with different concentrations of the common priming agent salicylic acid [38] 2 h before elicitation with ulvan (Fig. 3a). A low ulvan concentration (0.1 mg/ml) was used for elicitation, producing no first peak and only a small second peak in the absence of the priming agent, for optimal conditions to identify the priming effect. As the concentration of salicylic acid increased, the double burst started to show. Priming was evident for both peaks and pronounced dose dependency was observed starting at a salicylic acid concentration of ~0.7 µM and reaching saturation at ~5.5 µM (Fig. 3b).

**Fig. 4:**
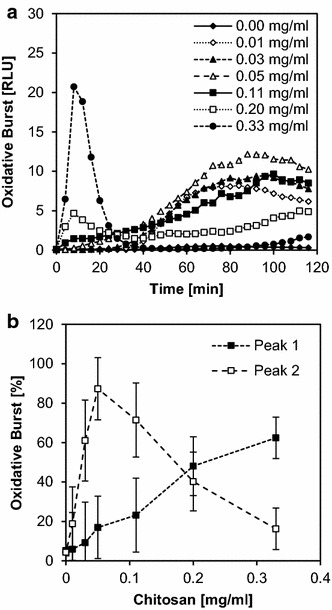
Elicitor dose dependency in the monocot *Oryza sativa.* Oxidative burst of *Oryza sativa* cell suspension cultures elicited with different concentrations of chitosan (0.01–0.33 mg/ml) in triplicates. During the 120-min measuring period, a first peak with high amplitude and short duration was observed followed by a second peak with longer duration (**a**). Each peak maximum was used to plot dose-dependency curves, in which peak 1 is defined as 0–20 min and peak 2 as 25–100 min (**b**). The data shown in **a** are taken from one experiment which is representative of ten independent experiments. The data shown in **b** are mean values ± SD of these ten experiments with the maximum relative light units (RLUs) reached in each experiment set to 100 %

Having optimized the method for dicot cells, we then transferred the same principles to a monocot system, i.e., rice (*Oryza sativa*) cell suspension cultures. These cells produce a less intense oxidative burst signal so the addition of 8 U/ml horseradish peroxidase as a catalyst was necessary. (In the case of *M. truncatula*, addition of peroxidase was not required but also did not disturb the assay; it amplified the first peak tenfold but not the second peak). Rice cells were pre-incubated in assay medium for 4 h before simultaneously adding L012, peroxidase, and the elicitor which in this case was chitosan as ulvan does not show elicitor activity in monocot plants. The addition of L012 itself did not elicit light emission in this system but the measurement became less reproducible when L012 was added to the medium during pre-incubation. Therefore, *O. sativa* cells were not pre-incubated with L012 in contrast to *M. truncatula* cells. As seen in the dicot system, the burst in rice cells showed two peaks within the 90-min measurement period. Again, the first peak had a high amplitude but was of short duration, and it was observed only at high concentrations of chitosan (0.2–0.3 mg/ml), reaching a maximum after 5–7 min before returning to the baseline after 20 min. The second peak reached a maximum at 60–80 min (Fig. 4a). Here, the maximal amplitude was not as high as for the first peak, but duration was more than four times increased. The dose dependency of the second peak (Fig. 4b) differed from that of the first peak, rapidly reaching an optimum concentration at ~0.05 mg/ml, then declining to zero activity at ~0.3 mg/ml. In contrast, the first peak increased steadily from a concentration of 0.05 mg/ml upwards.

**Fig. 5:**
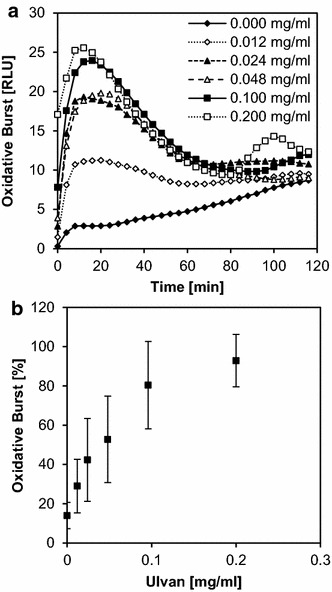
Priming dose dependency in the monocot *Oryza sativa.* Oxidative burst of *Oryza sativa* cell suspension cultures pre-treated with different concentrations of ulvan (0.012–0.2 mg/ml) in triplicates, then elicited with 0.08 mg/ml chitosan. During the 120-min measuring period, a first peak with high amplitude was observed sometimes followed by a second peak (**a**). The first peak maximum (0–30 min) was used to plot a dose-dependency curve (**b**). The data shown in **a** are taken from one experiment which is representative of ten independent experiments. The data shown in **b** are mean values ± SD of these ten experiments with the maximum relative light units (RLUs) reached in each experiment set to 100 %

Ulvan acts as an elicitor in dicot cells but shows priming activity in monocot cells [17]. When pre-treated with different concentrations of ulvan 2 h before measurement, this priming effect influenced the appearance of the first peak more than that of the second peak (Fig. 5a). The ulvan dose dependency of the first peak reached saturation at ~0.2 mg/ml (Fig. 5b).

**Fig. 6:**
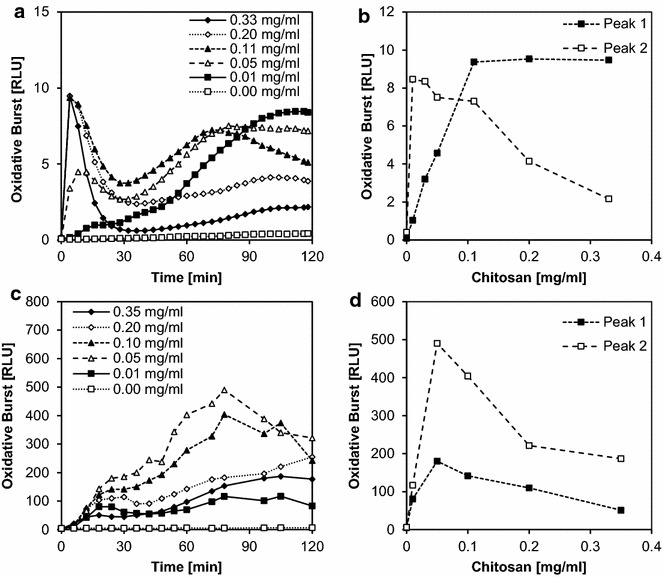
Direct comparison of cuvette-luminometer and micro titre plate luminometer based methods. **a**, **b** Oxidative burst in *Oryza sativa* suspension cultured cells using L012 as a chemiluminescent reagent in a 96 well plate elicited with different concentrations of chitosan (0.01–0.33 mg/ml) (data shown are means of triplicates). During the 120-min measuring period, a first peak with high amplitude and short duration was observed followed by a second peak with longer duration (**a**). Each peak maximum was used to plot dose-response curves, in which peak 1 is defined as 0–20 min and peak 2 as 25–100 min (**b**). **c**, **d** Oxidative burst in *O. sativa* suspension cultured cells using luminol as a chemiluminescent reagent in a cuvette, with different concentrations of chitosan as an elicitor (0.01–0.35 mg/ml). During the 120-min measuring period, a peak reached around 90 min was preceded by a shoulder which was defined as “peak 1” (**c**). Dose-response curves were plotted, in which peak 1 is defined as 0–20 min and peak 2 as 25–100 min (**d**). All plots represent one out of three independent experiments which gave similar results

To allow a direct comparison between the former and the improved method, the monitoring of ROS was performed in parallel in both systems with *O. sativa* cells (Fig. 6). In the microtiter plate reader, two peaks were again noticed as described before (Fig. 6a), and the dose response curves for the two peaks again differed. Here, peak 1 reached saturation at 0.11 mg/ml. Peak 2 showed a steep increase, reaching output maximum already with the lowest dose tested (0.01 mg/ml) and decreasing with higher concentrations (Fig. 6b). In the cuvette-luminometer, the ROS peak was reached at around 90 min for the high concentrations tested, showing a shoulder within the first 30 min (Fig. 6c). Interpretation of this shoulder as a first peak led to two dose response curves (Fig. 6d), both reaching a maximum at 0.05 mg/ml. A H_2_O_2_ calibration curve was recorded for both systems (Additional file 3).

## Discussion

We developed a microtiter plate screening assay using the chemiluminescent probe L012 for the continuous measurement of the oxidative burst in plant cell suspension cultures, allowing us to determine the dose dependencies of substances with even low elicitor and/or priming activities. A direct comparison between the microtiter plate versus the cuvette luminometer assay showed that both gave similar results, but the new method has higher time resolution and allows medium throughput measurements. Whereas established methods based on leaf discs have been impossible to adapt for monocot systems, we found that the cell-based assay worked with both dicot and monocot cells. The new method therefore allows the direct comparison of oxidative burst reactions induced by elicitors in monocot and dicot plants.

The ability to compare dicot and monocot systems directly is highly relevant given recent reports describing fundamental differences in the signaling molecules underlying immune surveillance. For example, oligogalacturonides act as endogenous elicitors or DAMPs in dicots [39] but not in monocots [40]. Furthermore, dicots and monocots appear to recognize different oligoglucans derived from fungal cell walls as PAMPs [41] and there are distinct pathways for the perception of chitin [42]. Bacterial exopolysaccharides [24] and the green algal polysaccharide ulvan act as elicitors in dicots [43] but as priming agents in monocots [17]. A more detailed understanding of innate immunity in monocot crops would be valuable given the dependence of much of the global human population on cereal crops.

Several parameters were carefully optimized to establish a reliable protocol for the sensitive and quantitative detection of ROS. We anticipated a linear increase in the generation of ROS with increasing cell density, but the oxidative burst appeared to be largely independent of the cell density over the range 0.01–0.03 g/ml, with a substantial decline in the response both above and below this range. This optimum cell density is four times lower than that in the original method [17] showing that the new system is highly sensitive to overcrowding, possibly due to the smaller volume and even smaller surface area available for ventilation.

Our experiments revealed an early double burst that was observed in both the dicot and the monocot cell suspension cultures. A biphasic oxidative burst with an initial non-specific peak within 2 h followed by a second burst with a maximum after 4–6 h has been observed in several plant species following bacterial inoculation leading to cell death, and was shown to depend on a *hypersensitive response and pathogenicity* gene cluster [3, 44, 45]. A similar biphasic burst with a first peak after ~30 min followed by a second peak after 3–5 h was reported for the crude cell wall elicitors of *Phytophthora sojae* in parsley cell suspension cultures [46]. However to our knowledge, a biphasic burst within the first 2 h after elicitation has not been described before. It is possible that continuous exposure to L012 or pH change in the medium during the measurement period simply causes the second burst to occur sooner, thus reducing the delay between the two bursts.

But perhaps more likely, the appearance of the double peak in all experiments could also reflect the different properties of luminol and L012. Low concentrations of luminol combined with potassium ferricyanide detect H_2_O_2_ [21] whereas only high concentrations of the reagent also allow to detect superoxide [47]. The original assay system used low concentrations of luminol and is therefore likely to detect H_2_O_2_ only. In contrast, L012 was originally reported to detect superoxide [48] and was later shown to also detect OH^•^ [49] and H_2_O_2_ [34]. The biphasic burst observed in our experiments may therefore reflect the detection of multiple ROS, e.g., first superoxide, followed by hydrogen peroxide produced from the superoxide by the action of plant superoxide dismutases. Verification of this hypothesis would require the individual identification of the different ROS, but was not the subject of this method development. The ability to detect multiple ROS combined with the higher resolution (more data points per time) which reveals the distinct double burst shows the new method to be more sensitive to screen for an oxidative burst compared to current methods. The direct comparison between the two methods revealed that the single peak observed in the cuvette assay corresponds to the second peak of the biphasic curve observed in the microtiter assay.

The behavior of the dicot and monocot systems investigated was similar but not identical. The differences may be species-dependent, in which case a broader panel of cell lines should be tested, or they may reflect the use of different elicitors in each system. Chitosan appears to be less active (or even toxic) at higher concentrations, whereas ulvan appears to show concentration-dependent elicitor activity at least over the concentration range we tested. Additionally, chitosan and ulvan are both high-molecular-weight polysaccharides and are probably cleaved and processed by enzymes secreted by the plant cells before or during the oxidative burst. Rice cells e.g., show secretion of chitinases [50], which may cleave chitosan as well. The delayed appearance of the processed molecules may trigger the second burst, whereas the unprocessed molecules may be responsible for the first. Additionally, it cannot be fully excluded that one of the peaks may be due to a second contaminating compound as even purified and well characterised biopolymers like the ulvan and chitosan fractions used here could carry small amounts of contaminations.

## Conclusion

We have developed a method for the reliable measurement of the oxidative burst in plant suspension cultured cells using a miniaturized format that allows medium-throughput screening for potential elicitors or priming agents. The method is sensitive enough to detect even weak elicitors, potentially allowing to screen for lead compounds that can then be developed into new biologics for agricultural plant protection, again using our assay to monitor the optimization process. We found that the method requires only minor adjustments when transferred to different cell systems and can therefore be applied in a broad variety of experimental settings. The simultaneous monitoring of ROS and Ca^2+^ signaling could be achieved using cell cultures expressing aequorin [51] in medium supplemented with L012 or coelenterazine in parallel wells within a single microtiter plate, thus providing an interesting combination of read-outs that may help to dissect the nature of the double burst. The microtiter format generally facilitates the addition of further assay readouts, e.g., colorimetric detection of residual elicitor, staining for cell viability, or enzyme-linked immunosorbent assays using specific antibodies. Such experiments carried out in parallel on the same batch of cells would provide further insight into the signaling pathways that are activated during plant defense responses.

## Methods

### Cultivation of cells

Four replicate lines of suspension cultured *M. truncatula* cells (initial line kindly provided by Prof. Karsten Niehaus, University of Bielefeld) were cultivated in the dark at 26 °C in 15 ml MS medium [52] supplemented with 45 µM 2,4–dichlorophenoxyacetic acid (2,4-D), 4.6 µM kinetin and 87.64 mM sucrose (pH 5.7), shaking at 120 rpm. Cells were transferred to fresh medium every 7 days.

*Oryza sativa* cells were cultivated in three replicate lines as previously described [13] on a rotary shaker (120 rpm) in the dark at 26 °C in 50 ml culture medium. The culture medium contained the following components (mM): KNO_3_ (27.99); NH_4_H_2_PO_4_ (3.5); CaCl_2_ (1.49); MgSO_2_ (0.86); FeSO_4_ (0.1); ethylenediamine-N,N,N′,N′-tetraacetic acid, disodium salt (0.1); MnSO_4_ (0.015); ZnSO_4_ (0.005); KI (0.005); H_3_BO_3_ (0.026); CuSO_4_ (0.0001); Na_2_MoO_4_ (0.001); CoCl_2_ (0.0001); nicotinic acid (0.004); thiamine-HCl (0.003); pyridoxine–HCl (0.002); myo-inositol (0.56); glycine (0.03); 2,4-dichlorophenoxyacetic acid (0.005); sucrose (87.67), and the pH was adjusted to 5.8 with KOH. Cells were transferred to fresh medium every 7 days and sieved through a mesh to make fine aggregates every 2 weeks.

The assay medium for *M. truncatula* cells comprised 4 % cultivation medium supplemented with 87.67 mM sucrose. The *O. sativa* assay medium was prepared similarly comprising 5 % cultivation medium and 87.67 mM sucrose. It was complemented with 10 mM MES and the pH was adjusted to 5.8.

### Elicitors and priming agents

Ulvan was extracted from *Ulva fasciata* as previously described [17] and its molecular weight of ~600,000 g/mol was confirmed by high-performance size exclusion chromatography (Agilent Technologies, Santa Clara, USA) on three PSS^®^ Suprema columns (one 100 Å guard column and two 3000 Å columns with an internal diameter of 8 mm) coupled to a refractive index detector (Agilent series 1200 RID). The molecular weight was determined by calibration using a series of pullulans (PSS, Mainz, Germany). The validity of calibration for negatively-charged polysaccharides was confirmed using dextran sulfates (Sigma–Aldrich, Taufkirchen, Germany).

The chitosan used in our experiments was produced from commercial chitosan (Sigma–Aldrich) as previously described [53]. The average degree of polymerization was 400 (determined by high-performance size exclusion chromatography with refractive index detection and multi-angle laser light scattering as well as online viscosimetry) and the average degree of acetylation was 14 % (determined by proton nuclear magnetic resonance spectroscopy).

Salicylic acid and horseradish peroxidase (Typ II) were purchased from Sigma–Aldrich, and L012 was obtained from Wako Chemicals (Neuss, Germany).

### Measuring the oxidative burst

#### In a microtiter plate reader

Cells were used to monitor the oxidative burst 3–4 days after transfer to new medium. After removing the culture medium, *M. truncatula* and *O. sativa* cells were weighed and diluted in the assay medium at different cell densities. The cell suspension cultures were then transferred to a white 96 U-Nunc plate (Thermo Fisher Scientific, Waltham, MA) using a multichannel pipette and wide-bore tips (200 µl per well). We added fresh L012 solution (0.3 mM dissolved completely in 50 mM potassium phosphate buffer, pH 7.9) to the *M. truncatula* cultures. The plates were incubated as described above for 4 h (unless stated otherwise) in a cell culture chamber or in the luminometer (see below), with similar results. For priming experiments, the priming agent was added 2 h before elicitation.

After incubation, the oxidative burst was measured using a luminometer (Luminoskan Ascent, Thermo Fisher Scientific) which added reagents automatically. The *O. sativa* cell cultures were supplemented with L012 (0.03 mM) and horseradish peroxidase (8 U/ml) directly before measurement, if not stated otherwise. For both cell lines, different concentrations of elicitor were added by automatically applying different volumes of elicitor stock solution (1 mg/ml). Measurements were taken for 1 s per well every 5 min for *M. truncatula* and every 2 min for *O. sativa* (but only every second data point was plotted) over a measuring period of 90 min unless otherwise stated. At least triplicates were taken for each sample per experiment.

#### In a cuvette luminometer

Three to four day old cells were diluted in assay medium at a concentration of 60 mg/ml and incubated in 6-well plates (5 ml/well) for 5 h shaking at 120 rpm in the dark at 26 °C. At different time points after elicitation, aliquots of 200 µl were added to 700 µl of 50 mM potassium phosphate buffer, pH 7.9, and light detection was performed in a cuvette luminometer (Lumat LB 9501, Berthold, Bad Wildbach, Germany) after automatic addition of 100 μl luminol (Sigma; 1.21 mM in phosphate buffer) and 100 μl 14 mM potassium hexacyanoferrate (Fluka), using an integration time of 10 s, at 430 nm.

### Statistical analysis

To test the curve progression of concentration dependency of the two peaks for significance, a Mann–Whitney rank sum test was implemented in Sigma plot 12 for all data points. To deduce from the data points to the curve, the combined p value was determined using χ^2^ and a degree of freedom (df) twice the number of p values with Excel 7 and the function chidist (χ^2^, df). p values smaller than 0.05 were interpreted as significantly different.

$$\chi^{2} = - 2\sum\nolimits_{i = 1}^{c} {{ \ln }\;\left( p \right)}$$

## Additional files

10.1186/s13007-016-0110-1 Initial measurement of elicitor dose dependency in the dicot *Medicago truncatula.* Dose dependency of the oxidative burst in *Medicago truncatula* cell suspension cultures elicited with different concentrations of ulvan (0.02–0.2 mg/ml) (n = 12). During the 70-min measuring period, first a peak with high amplitude and short duration was observed followed by a second peak with lower amplitude and longer duration (A). The data shown in (B) show the slope between first two measurement points. Slope was determined for all 12 repetitions. Here mean and standard deviation are plotted against ulvan concentration.
10.1186/s13007-016-0110-1 Catalase. Oxidative burst in *Medicago truncatula* suspension cultured cells elicited with different doses of ulvan (0.1 mg/ml; 0.05 mg/ml) (data shown are means of duplicates with (black symbols) and without (white symbols) addition of 175 U/ml catalase (from bovine liver; Sigma–Aldrich, Taufkirchen, Germany). The plots represent one out of three independent experiments which gave similar results.
10.1186/s13007-016-0110-1 Calibration curve. Calibration of RLU readings using H_2_O_2_ in 96 well plate reader with L012, peroxidase and heat-inactivated rice cells in rice cell assay medium (n = 7) (A). Linear regression gave a R-square value of 0.99 (Excel). H_2_O_2_ calibration curve in cuvette luminometer with luminol and KHCF in rice cell assay medium (n = 6) (B). Linear regression gave a R-square value of 0.95 (Excel).
