# Supplementary figures and images for: An improved microtiter plate assay to monitor the oxidative burst in monocot and dicot plant cell suspension cultures
Source: Plant Methods. 2016 Jan 26;12:5. doi: 10.1186/s13007-016-0110-1 (PMC4729151; doi:10.1186/s13007-016-0110-1)

## Slide 1
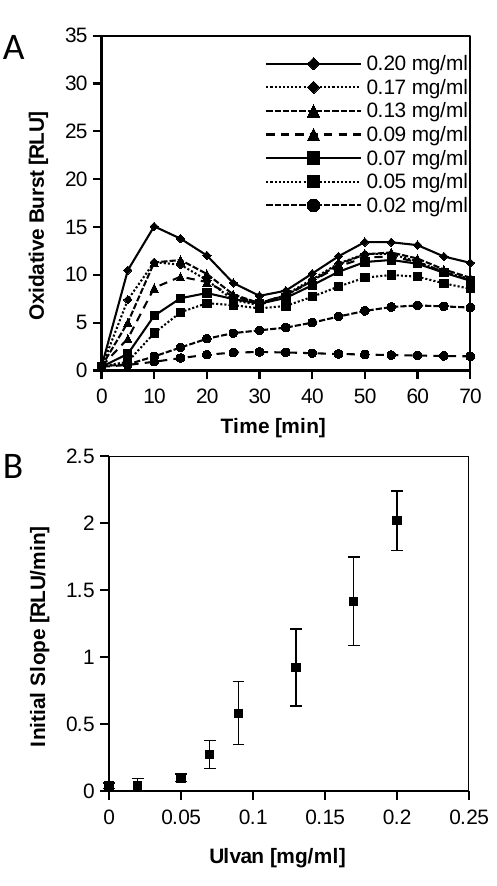

### Chart
| Category | 0.20 mg/ml | 0.17 mg/ml | 0.13 mg/ml | 0.09 mg/ml | 0.07 mg/ml | 0.05 mg/ml | 0.02 mg/ml | 0.00 mg/ml |
|---|---|---|---|---|---|---|---|---|A
B
### Chart
| Category | |
|---|---|

Supplement: Supplementary file 1 — 10.1186/s13007-016-0110-1 Initial measurement of elicitor dose dependency in the dicot Medicago truncatula. Dose dependency of the oxidative burst in Medicago truncatula cell suspension cultures elicited with different concentrations of ulvan (0.02–0.2 mg/ml) (n = 12). During the 70-min measuring period, first a peak with high amplitude and short duration was observed followed by a second peak with lower amplitude and longer duration (A). The data shown in (B) show the slope between first two measurement points. Slope was determined for all 12 repetitions. Here mean and standard deviation are plotted against ulvan concentration. [file 13007_2016_110_MOESM1_ESM.pptx]

## Slide 1
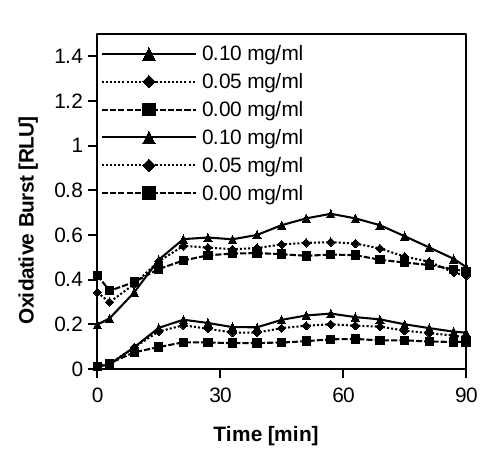

### Chart
| Category | 0.10 mg/ml | 0.05 mg/ml | 0.00 mg/ml | 0.10 mg/ml | 0.05 mg/ml | 0.00 mg/ml |
|---|---|---|---|---|---|---|

Supplement: Supplementary file 2 — 10.1186/s13007-016-0110-1 Catalase. Oxidative burst in Medicago truncatula suspension cultured cells elicited with different doses of ulvan (0.1 mg/ml; 0.05 mg/ml) (data shown are means of duplicates with (black symbols) and without (white symbols) addition of 175 U/ml catalase (from bovine liver; Sigma–Aldrich, Taufkirchen, Germany). The plots represent one out of three independent experiments which gave similar results. [file 13007_2016_110_MOESM2_ESM.pptx]

## Slide 1
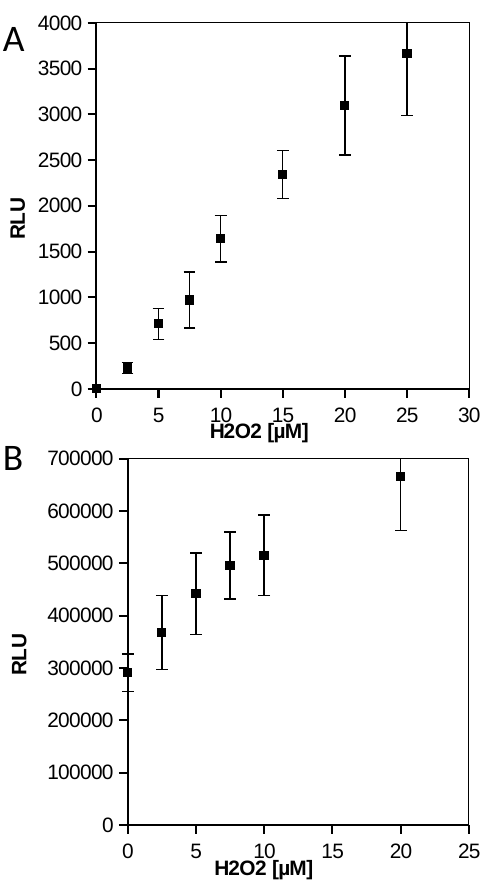

### Chart
| Category | |
|---|---|A
B
### Chart
| Category | |
|---|---|

Supplement: Supplementary file 3 — 10.1186/s13007-016-0110-1 Calibration curve. Calibration of RLU readings using H2O2 in 96 well plate reader with L012, peroxidase and heat-inactivated rice cells in rice cell assay medium (n = 7) (A). Linear regression gave a R-square value of 0.99 (Excel). H2O2 calibration curve in cuvette luminometer with luminol and KHCF in rice cell assay medium (n = 6) (B). Linear regression gave a R-square value of 0.95 (Excel). [file 13007_2016_110_MOESM3_ESM.pptx]
